# SIRT6 ameliorates LPS-induced apoptosis and tight junction injury in ARDS through the ERK1/2 pathway and autophagy

**DOI:** 10.7150/ijms.80920

**Published:** 2023-03-05

**Authors:** Hanhan Liu, Sijiao Wang, Linjing Gong, Yue Shen, Fan Xu, Yali Wang, Lijuan Hu, Lei Zhu

**Affiliations:** 1 Department of Pulmonary and Critical Care Medicine, Zhongshan Hospital, Fudan University, Shanghai 200032, China; 2 Department of Respiratory and Critical Care Medicine, West China Hospital, Sichuan University, Chengdu, Sichuan 610041, China; 3 Department of Respiratory and Critical Care Medicine, Huadong Hospital, Fudan University, Shanghai 200040, China

**Keywords:** Acute respiratory distress syndrome (ARDS), Sirtuin6, tight junction, ERK1/2, OSS_128167, autophagy

## Abstract

Sirtuin6 (SIRT6) has been demonstrated to be involved in a range of physiological processes and diseases, while its role in acute respiratory distress syndrome (ARDS) remains unclear. Therefore, this study focused on the role and underlying mechanism of SIRT6 in ARDS with the aim of identifying potential therapeutic targets. In this study, we found that SIRT6 was significantly decreased in lipopolysaccharide (LPS)-induced A549 cells and a murine model. *In vitro* overexpression of SIRT6 restored the expression of tight junction proteins (ZO-1 and occludin) and alleviated cell apoptosis and inflammation, while knockdown of SIRT6 aggravated the loss of tight junction proteins (ZO-1 and occludin) and promoted cell apoptosis and inflammation in LPS-induced A549 cells. Furthermore, the overexpression of SIRT6 enhanced autophagy and inhibited the ERK1/2 pathway, while the knockdown of SIRT6 inhibited autophagy and activated the ERK1/2 pathway. The autophagy activator rapamycin and the ERK1/2 inhibitor PD98059 rescued the effects of SIRT6 knockdown on tight junction proteins, apoptosis, and inflammation. Mechanistically, SIRT6 deacetylated histone 3 at Lys9 to negatively regulate the ERK1/2 pathway. *In vivo*, the SIRT6-specific inhibitor OSS_128167 also significantly accelerated LPS-induced loss of tight junction proteins, lung inflammation, and apoptosis. Meanwhile, the SIRT6-specific inhibitor OSS_128167 also activated the ERK1/2 pathway and inhibited lung autophagy. These results suggested that SIRT6 could ameliorate the loss of tight junction proteins, inflammation, and apoptosis in LPS-induced ARDS by inhibiting the ERK1/ 2 pathway and enhancing autophagy, indicating that SIRT6 plays a beneficial role in ARDS and might be a potential therapeutic target for ARDS.

## Introduction

Acute respiratory distress syndrome (ARDS) is a clinical syndrome characterized by progressive dyspnea and refractory hypoxemia. The pathophysiology changes in ARDS include damage of the pulmonary microvascular endothelium and alveolar epithelium (caused by uncontrolled inflammation) and increased permeability of the alveolar epithelial-endothelial barrier to fluid, proteins, and neutrophils, resulting in pulmonary edema 1. ARDS has a high morbidity and mortality rate, and its mechanism is still unclear. According to a previous study, nearly 10% of patients in intensive care units fulfilled the criteria for ARDS 2, with the mortality rate ranging from 31 to 45% 2-4. There is currently no effective pharmacotherapy for ARDS. Bacterial or viral infections are the most common triggers. Lipopolysaccharide (LPS), a key component of bacterial endotoxin, has been widely used to induce ARDS models 5. Elucidating the molecular mechanism of LPS-induced ARDS could provide a foundation for the clinical treatment of ARDS.

Inflammation, apoptosis, and disruption of the lung epithelial-endothelial barrier are common injuries in ARDS. Tight junctions (TJs) are found in the most apical region of cell-cell contacts and are composed of cytoplasmic proteins such as (Zona occludens) ZO-1, ZO-2, and ZO-3, and a family of transmembrane proteins (claudins, occludin, and junctional adhesion molecule) 6. Among them, ZO-1 binds to intracellular skeletal proteins, and occludin binds directly to ZO-1. Both proteins are crucial for maintaining TJ function. TJs serve as a permeability barrier to prevent molecules from freely diffusing between cells 6, 7. The disruption of these proteins is linked to dysfunction of the lung epithelial-endothelial barrier 8, 9. In ARDS, an excessive inflammatory response leads to barrier dysfunction by disrupting the expression of intercellular TJ proteins and increasing apoptosis and necrosis 1, 10. Pulmonary barrier dysfunction further aggravates inflammatory cell infiltration, triggering inflammatory storms and exacerbating ARDS progression. Therefore, controlling inflammation, reversing apoptosis, and repairing the expression of intercellular TJ proteins are effective strategies for ARDS treatment.

Autophagy is a self-degrading mechanism that selectively targets dysfunctional organelles, intracellular microorganisms, and pathogenic proteins, and the defects in autophagy can result in various diseases 11, 12. The role of autophagy in ARDS is controversial. However, more and more studies have shown that autophagy plays a protective role in ARDS. For instance, penehyclidine hydrochloride can increase BEAS-2B cell autophagy and thus reduce apoptosis and inflammation in ARDS 13. Previous studies have shown that autophagy activation could increase the intestinal epithelial barrier and TJ protein levels 14. The role of autophagy in regulating TJ proteins in LPS-induced ARDS remains unclear.

Sirtuin6 (SIRT6), a member of the SIRT1-SIRT7 family, is a highly conserved NAD^+^-dependent deacetylase that was first identified in *Saccharomyces cerevisiae* and is located mainly in the nucleus 15, 16. It can bind to chromatin to deacetylate histone H3 in the target genes' promoter regions. Thus, SIRT6 regulates gene transcription and participates in several pathophysiological processes. It can reduce inflammatory responses, repair DNA damage, inhibit apoptosis, and contribute to antioxidant stress 17, 18. SIRT6 activates autophagy in a variety of cells 19-21. However, the role of SIRT6-related autophagy in ARDS is rarely studied. The extracellular signal-regulated kinases (ERK1/2 MAPK) regulate many processes, including cell adhesion, cell migration, cell survival, cell differentiation, and transcription 22. The ERK1/2 pathway is also involved in regulating pulmonary inflammation, apoptosis, and TJ proteins 23, 24. A recent study found that SIRT6 deacetylates H3K9 in the ERK1/2 promoter region to inhibit the ERK1/2 pathway and reduce renal damage 25. Given this, whether SIRT6 regulates inflammation, apoptosis, and TJ protein expression by regulating the ERK1/2 pathway and autophagy in ARDS was unclear. Therefore, it was necessary to clarify the above role of SIRT6 and elucidate the role and mechanism of SIRT6 in ARDS.

In this study, we hypothesized that SIRT6 could inhibit the ERK1/2 pathway by deacetylating H3K9 and enhance autophagy to improve inflammation, apoptosis, and the expression of TJ proteins in LPS-induced ARDS. In this study, LPS-induced A549 cells and murine models were used to clarify the role and potential mechanism of SIRT6 in ARDS. This study hopes to provide new light on the molecular pathogenesis of ARDS and potential therapeutic targets for ARDS.

## Materials and methods

### Cell culture and drug treatment

Human type II alveolar epithelial A549 cells were purchased from the Chinese Academy of Sciences Cell Bank (Shanghai, China). The A549 cells were cultured in RPMI-1640 medium supplemented with 10% fetal bovine serum (Gibco, Grand Island, NY, USA), and maintained in a humidified incubator at 37 °C with 5% CO_2_. LPS from *Escherichia coli* O111:B4 (#L2630, Sigma-Aldrich, Saint Louis, MI, USA) was dissolved in sterile phosphate-buffered saline (PBS), and A549 cells were stimulated with different concentrations of LPS for 24 h or with 5 µg/mL LPS for different periods. The control group was stimulated with the same volume of PBS. PD98059 (20µM) and rapamycin (200nM) were added to the cells 1 h before LPS stimulation, and the cells were harvested 24 h later for further experiments. Cells in all of the experiments were within 20 generations. All the experimental results were confirmed through at least three independent cultures of A549 cells.

### Cell transfection

SIRT6 siRNA, negative control (si-NC), SIRT6 overexpression plasmid, and vector were designed and synthesized by GenePharma (Shanghai, China). A549 cells were seeded in 6-well plates. When the cell density reached 40%-50%, siRNA or overexpression plasmid was transfected into the cells according to the manufacturer of Lipofectamine 3000 (Invitrogen, Carlsbad, CA, USA). The culture medium was changed after 24 h and the cells were collected for detection 48 h after transfection.

### Quantitative real-time reverse transcriptase- PCR (qRT-PCR)

PrimeScript^TM^ RT Master Mix was used to reverse-transcribe RNA into cDNA (#RR036A, Takara, Kyoto, Japan). The qRT-PCR reactions on cDNA were carried out using the Applied Biosystems 7500HT Real-Time PCR System (Foster City, CA, USA) and Hieff® qPCR SYBR Green Master Mix (Low Rox Plus; YEASEN, Shanghai, China). The relative target genes' mRNAs were measured using the 2^-ΔΔCt^ technique and GAPDH for normalization as per the manufacturer's instructions for the procedure. The primers used for qRT-PCR are shown in Table 1.

### Western blot analysis

Proteins were isolated using RIPA lysate buffer. Protein concentrations were determined by the BCA protein assay kit (Beyotime, Shanghai, China). Protein samples were separated by SDS-PAGE and transferred to polyvinylidene difluoride (PVDF) membranes (Millipore, Billerica, MA, USA). After blocking with 5% BSA for 1 h at room temperature, the membranes were incubated with primary antibodies overnight at 4 °C and then incubated with the secondary antibodies for 1 h at room temperature. Finally, the immunoblot was visualized by a gel densitometric scanning system and quantified using ImageJ (National Institutes of Mental Health, Bethesda, MD, USA).

The primary antibodies were as follows: SIRT6 (#ab191385; abcam; 1:1000), H3K9Ac (#ab32129; abcam; 1:1000), ERK1/2 (#4695S; CST; 1:1000), p-EKR1/2 (#9101S; CST; 1:1000), acetylated-lysine antibody (#9441S; CST; 1:1000), ZO-1 (#61-7300; Invitrogen; 1:500 and #ab276131; abcam; 1:1000), occludin (#ab216327; abcam; 1:1000), GAPDH (#A19056; Abclonal; 1:2000), LC3B (#ab51520; abcam; 1:1000), Beclin1 (#A21191; Abclonal; 1:1000), and α-tubulin (#AF0001; Beyotime; 1:1000).

### Immunofluorescence

A549 cells were fixed with 4% paraformaldehyde for 15 min and then blocked for 30 min with 5% BSA. The cells were first treated with primary antibodies against ZO-1 and occludin overnight at 4 °C, followed by 1 h at room temperature with fluorescein isothiocyanate-conjugated secondary antibodies (Alexa Fluor 488 IgG, Beyotime). Samples were captured using a confocal microscope (Nikon, Japan).

Lung tissues were sectioned into 5-μm-thick slices, deparaffinized with xylene, and then dehydrated with an ethanol gradient. After thermal repair of the antigens, the samples were blocked with goat serum for 30 min at room temperature and then incubated overnight at 4 ℃ with primary antibodies against ZO-1 and occludin. The slides were incubated with fluorescein isothiocyanate-conjugated secondary antibodies (Alexa Fluor 488/Cy3 IgG, Beyotime) for 1 h at room temperature. The sections were incubated with 4′,6-diamidino-2-phenylindole (DAPI) (#D8417, Sigma-Aldrich) for 8 min, and the slices were sealed with a fluorescence decay-resistant medium. The samples were imaged with a fluorescence microscope (Nikon).

### Detection of cell apoptosis

Cells were collected, twice-washed with PBS, resuspended in a binding buffer, and cultured with PI and annexin V‐FITC according to the manufacturer's instructions (Beyotime). Finally, a FACSAria II (BD Biosciences, New Jersey, America) was used to evaluate cell apoptosis instantly. A quantitative analysis was carried out using FlowJo X10.0.7r2 software (BD Biosciences).

### Co-immunoprecipitation

Co-immunoprecipitation (Co-IP) lysis buffer (50 mM Tris, pH 7.5; 150 mM NaCl; 5 mM EDTA; 5 mM EGTA; 1% NP40; 15 mM MgCl_2_6H2O) containing PMSF and phosphatase inhibitor was used to collect A549 cells. After centrifuging the lysate at 14000g for 10 min at 4 °C, the supernatant lysate was transferred to a fresh 1.5 mL EP tube. Protein A/G magnetic beads (#B23201, Bimake, USA) bound to an anti-SIRT6 antibody, an anti-ERK1/2 antibody, or normal rabbit IgG were incubated overnight at 4 °C with the supernatant lysates. By boiling the beads in a 2×loading buffer, proteins were eluted. Western blot was used to evaluate the materials.

### Chromatin immunoprecipitation assay

Chromatin immunoprecipitation (CHIP) experiments were performed using the SimpleChIP® Plus Enzymatic Chromatin IP Kit (#9005, CST) following the manufacturer's instructions. Briefly, samples were cross-linked for 10 min at room temperature with 1% formaldehyde before the reaction was stopped with glycine. Chromatin was digested to 150-900 bp using micrococcal nuclease enzymes. The antibody was added to the samples to form a DNA antibody complex. Complexes were precipitated with protein A/G magnetic beads and washed three times to remove non-specific DNA. Elution buffer was then added to elute the DNA. Cross-linking buffer was uncoupled for 2 h at 65 ℃, and DNA was then purified using a DNA purification column. The outcome of the qRT-PCR used to detect the target DNA was expressed as a percentage of the input.

### Animals and treatments

C57BL/6 male mice (6-8 weeks, weight 20-25 g) were purchased from Laboratory Animals (Shanghai, China). All mice were housed in the Animal Center at Zhongshan Hospital, Fudan University, under specialized pathogen-free conditions. This study was approved by the Animal Care and Use Committee of Zhongshan Hospital (2022-009).

The mice were divided randomly into four groups: the control group (n = 6), OSS_128167 group (n = 6), the LPS group (n = 6), and LPS+OSS_128167 group (n = 6). Mice were given an intraperitoneal injection of 1% sodium pentobarbital to induce anesthesia and then fixed to a 45-degree inclined board. LPS was dissolved in 0.9% normal saline and then instilled intratracheally at a dose of 5 mg/kg. To ensure that LPS was distributed evenly throughout the mouse lungs, the mice were placed in a prone position after 30 seconds of vertical rotation. Mice in the control group received the same volume of 0.9% saline and manipulations. To inhibit SIRT6, mice were intraperitoneally injected with 80 mg/kg OSS_128167 (Selleck, USA) 1 h before LPS administration. All mice were sacrificed 24 h after saline or drug administration, and the lungs and bronchoalveolar lavage fluid (BALF) were collected for further study.

### Histopathology

The lungs were fixed in 4% paraformaldehyde, embedded in paraffin, cut into 5-µm sections, and stained with hematoxylin and eosin (H&E) for histopathologic evaluation. The histopathology was assessed in a double-blind manner according to the following criteria 26: the presence of exudates, hyperemia or congestion, neutrophilic infiltrates, intra-alveolar hemorrhage or debris, and cellular hyperplasia. Each item was graded on a four-point scale from 0 to 3: 0 (normal lungs), 1 (mild injury), 2 (moderate injury), and 3 (severe injury).

### Analysis of bronchoalveolar lavage fluid

BALF was centrifuged at 300g for 5 min and 4 ℃. The supernatant was stored at -80 °C. Neutrophils were identified as CD45^+^Ly6G^+^CD11b^+^ when the cell sediments were resuspended in PBS and stained with FITC-anti-CD45, PerCP/Cyanine5.5-Ly6G, and APC/Cyanine7-CD11b (BioLegend, San Diego, CA, USA) 27. Total cells in the BALF were counted using a hemocytometer.

### Enzyme-linked immunosorbent assay (ELISA)

According to the manufacturer's protocols, cytokine levels in BALF were detected using an ELISA DuoSet kit (R&D Systems). Kits were used for measuring tumor necrosis factor-alpha (TNF-α) and IL-1β.

### Terminal deoxynucleotidyl Transferase-Mediated nick end labeling assay (TUNEL) assay

Apoptotic cells were detected *in situ* by TUNEL staining (Servicebio, Wuhan, China). A fluorescence microscope with 400× magnification (Nikon) was used to capture the images.

### Statistical Analysis

Data were expressed as the mean **±** standard deviation (SD) and analyzed with GraphPad Prism version 7.0 software (GraphPad Software Inc, USA). The differences between the two groups were assessed using the Student's *t*-test, and *p* < 0.05 was regarded as statistically significant.

## Results

### SIRT6 alleviates inflammation and enhances autophagy in LPS-induced A549 cells

**Figure 1:**
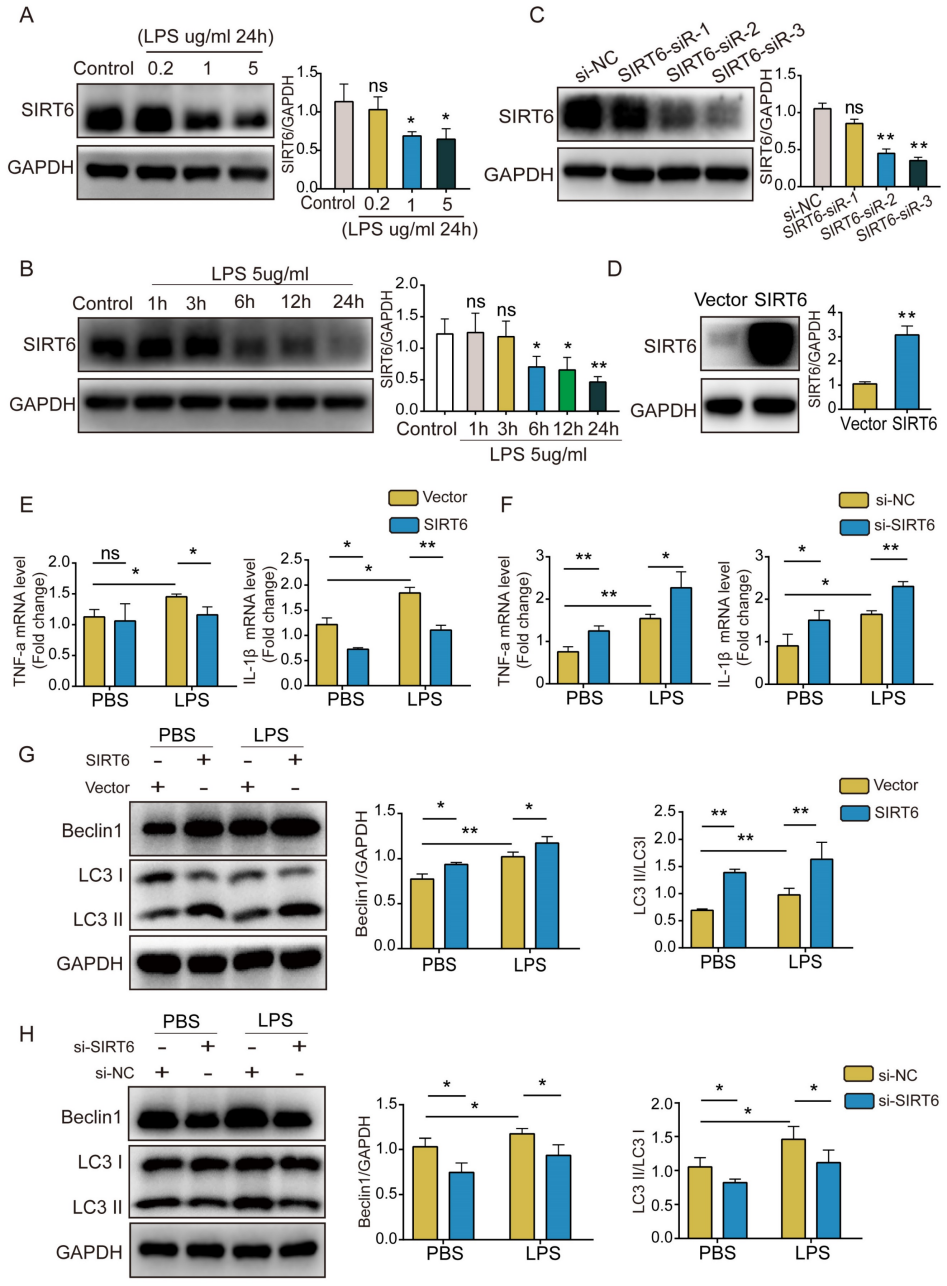
** SIRT6 alleviates inflammation and enhances autophagy in LPS-induced A549 cells.** (A and B) Representative western blot images and quantitative analysis of SIRT6 protein in A549 cells after treatment with different doses of LPS or LPS (5 μg/mL) for different times. (C) The transfection efficiency of SIRT6 siRNA was assessed by western blot in A549 cells. (D) The transfection efficiency of SIRT6 overexpression plasmid was assessed by western blot in A549 cells. (E) The mRNA levels of TNF-a and IL-1β were tested by qRT-PCR in LPS-induced A549 cells with SIRT6 overexpression. (F) The mRNA levels of TNF-a and IL-1β were tested by qRT-PCR in LPS-induced A549 cells with SIRT6 knockdown. (G) Representative Western blot images and quantitative analysis of autophagy-associated proteins in LPS-induced A549 cells with SIRT6 overexpression. (H) Representative Western blot images and quantitative analysis of autophagy-associated proteins in LPS-induced A549 cells with SIRT6 knockdown. All experiments were conducted in triplicate. Data are shown as the mean ± SD. The horizontal lines above bars indicate comparisons between groups, ns: no significance, *p<0.05, **p<0.01.

To better explore the role of SIRT6 in ARDS, we used LPS to stimulate A549 cells and establish a cell model. SIRT6 protein levels were determined by western blotting. The results showed that SIRT6 protein levels decreased in a concentration- and time-dependent way (Figure 1A and B). Subsequently, an siRNA strategy was used to silence the expression of SIRT6. Western blot analysis showed that SIRT6-siR-3 transfection significantly reduced the expression of the SIRT6 protein (Figure 1C). We used an overexpression plasmid to overexpress the SIRT6 gene in A549 cells. The overexpression efficiency of SIRT6 is shown in Figure 1D. qRT-PCR results showed that the levels of TNF-α and IL-1β were significantly increased after LPS stimulation, and overexpression of SIRT6 decreased TNF-α and IL-1β levels (Figure 1E). The knockdown of SIRT6 further increased TNF-α and IL-1β levels (Figure 1F). Meanwhile, we found that autophagy was activated in LPS-induced A549 cells, the overexpression of SIRT6 further enhanced autophagy, and the knockdown of SIRT6 inhibited autophagy (Figure 1G and H). These results suggest that SIRT6 is involved in regulating the inflammatory response and autophagy in ARDS.

### SIRT6 alleviates apoptosis and TJ injury in LPS-induced A549 cells

**Figure 2:**
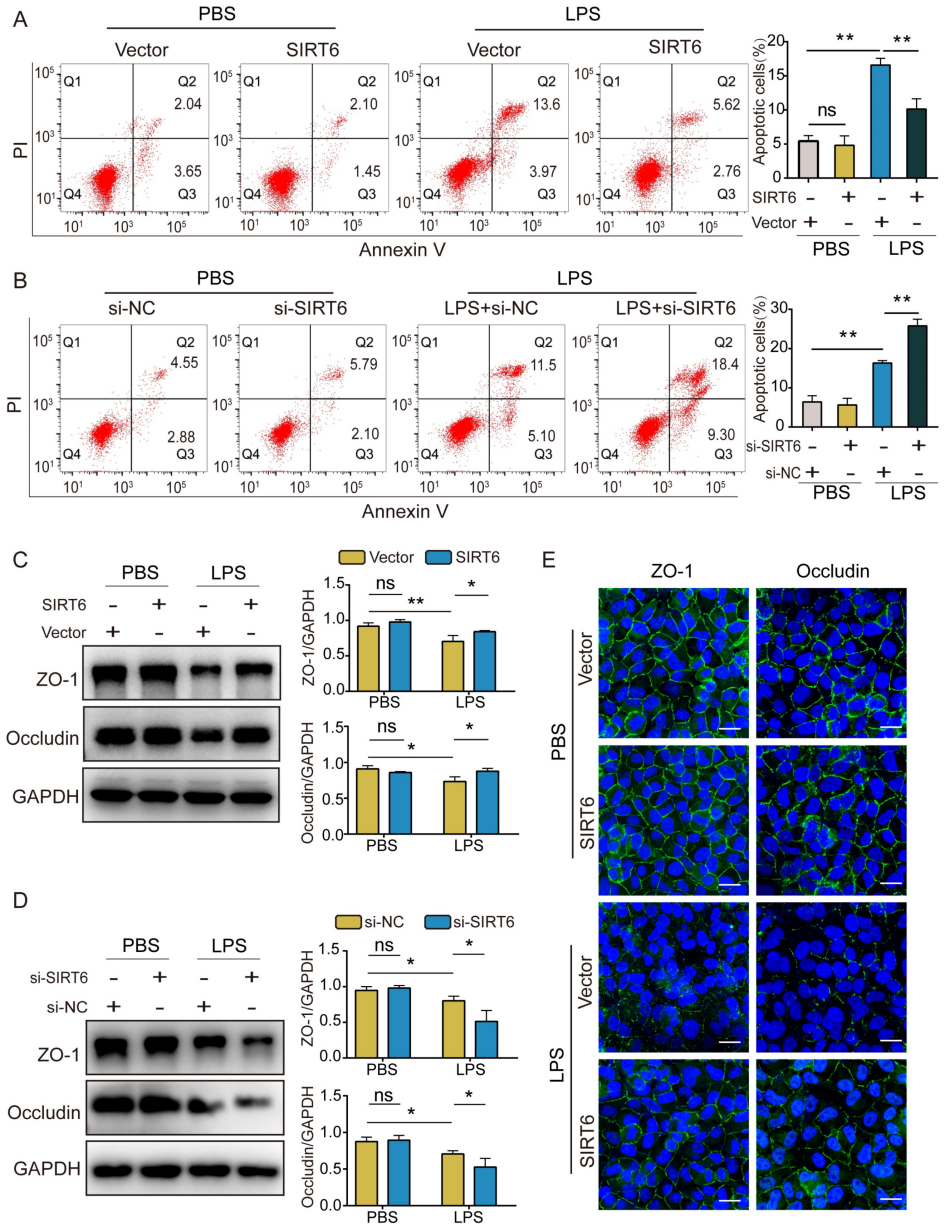
** SIRT6 alleviates apoptosis and TJ injury in LPS-induced A549 cells.** (A) The proportion of apoptotic cells was detected by flow cytometry in LPS-induced A549 cells with SIRT6 overexpression. (B) The proportion of apoptotic cells was detected by flow cytometry in LPS-induced A549 cells with SIRT6 knockdown. (C) Representative western blot images and quantitative analysis of TJ proteins (Zo-1, occludin) in LPS-induced A549 cells with SIRT6 overexpression. (D) Representative western blot images and quantitative analysis of TJ proteins (Zo-1, occludin) in LPS-induced A549 cells with SIRT6 knockdown. (E) Representative immunofluorescence staining of TJ proteins (Zo-1, occludin) in LPS-induced A549 cells with SIRT6 overexpression; Scale bar=20μm. All experiments were conducted in triplicate. Data are shown as the mean ± SD. The horizontal lines above bars indicate comparisons between groups, ns: no significance, *p<0.05, **p<0.01.

In this study, we investigated the effect of SIRT6 on apoptosis and TJ proteins. Flow cytometry demonstrated that apoptotic cells increased after LPS exposure compared with the control group. The overexpression of SIRT6 could reverse LPS-induced apoptosis (Figure 2A), while the knockdown of SIRT6 further aggravated LPS-induced apoptosis (Figure 2B). Similarly, western blot showed that the expression of ZO-1 and occludin proteins in LPS-induced A549 cells was significantly downregulated compared with the control group. The overexpression of SIRT6 could restore the expression of ZO-1 and occludin proteins (Figure 2C), while the knockdown of SIRT6 further impaired the expression of ZO-1 and occludin proteins (Figure 2D). Meanwhile, immunofluorescence showed that the overexpression of SIRT6 could restore the protein levels of ZO-1 and occludin in A549 cells (Figure 2E). These results suggest that SIRT6 protects the integrity of TJs and reduces cell apoptosis in ARDS.

### SIRT6 inhibits the ERK1/2 pathway through deacetylation of H3K9

**Figure 3:**
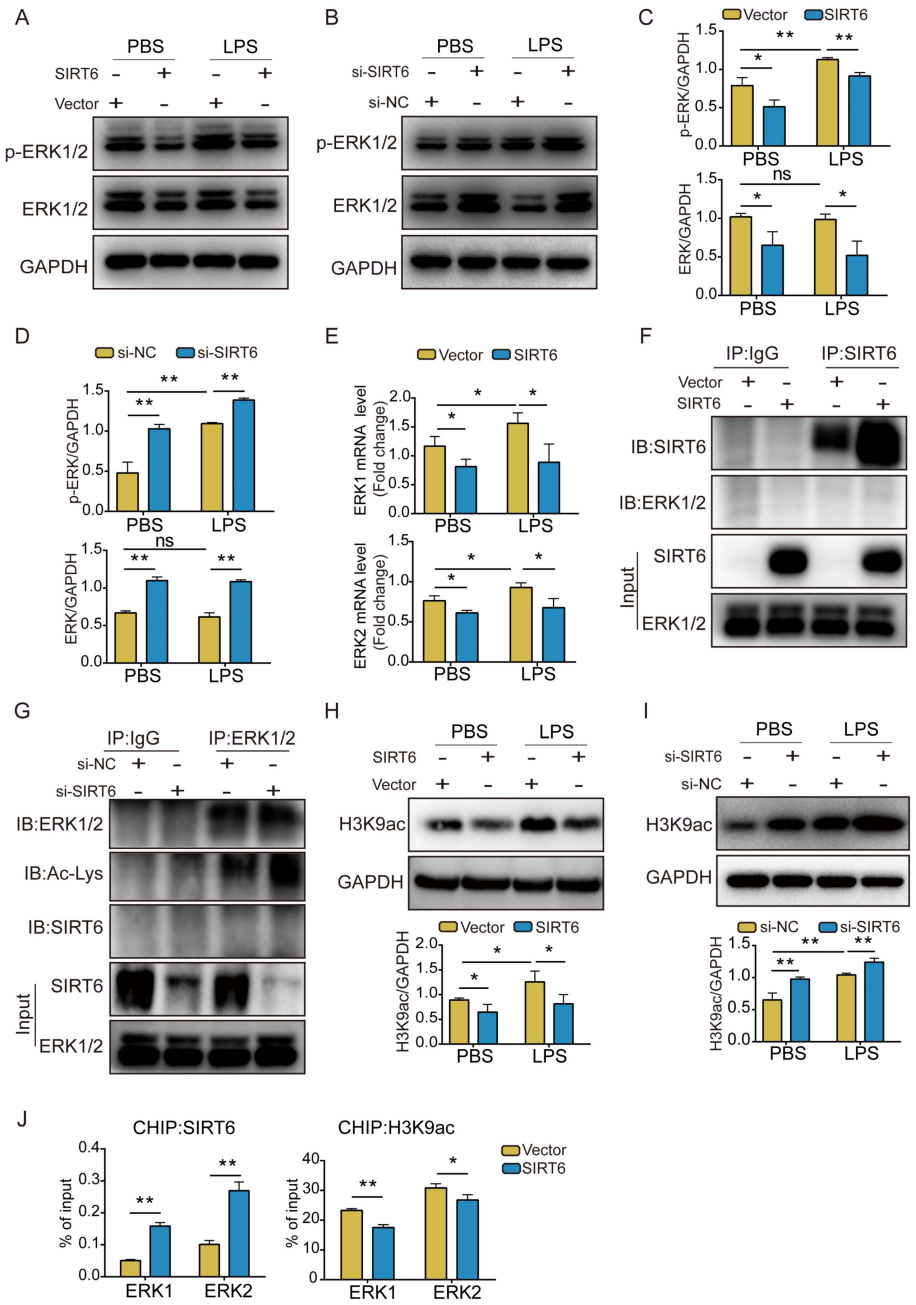
** SIRT6 inhibits the ERK1/2 pathway through deacetylation of H3K9.** (A) Representative western blot images of ERK1/2 and phosphorylated ERK1/2 (p-ERK1/2) proteins in LPS-induced A549 cells with SIRT6 overexpression. (B) Representative western blot images of ERK1/2 and phosphorylated ERK1/2 (p-ERK1/2) proteins in LPS-induced A549 cells with SIRT6 knockdown. (C) Quantitative analysis of ERK1/2 and phosphorylated ERK1/2 proteins in LPS-induced A549 cells with SIRT6 overexpression. (D) Quantitative analysis of ERK1/2 and phosphorylated ERK1/2 proteins in LPS-induced A549 cells with SIRT6 knockdown. (E) The mRNA levels of ERK1 and ERK2 were tested by qRT-PCR in LPS-induced A549 cells with SIRT6 overexpression. (F) Cell lysates were immunoprecipitated with IgG or anti-SIRT6 antibody and probed with the indicated antibodies in SIRT6-overexpression A549 cells. (G) Cell lysates were immunoprecipitated with IgG or anti-ERK1/2 antibodies and probed with the indicated antibodies in SIRT6-knockdown A549 cells. (H) Representative western blot images and quantitative analysis of acetylated H3K9 levels LPS-induced A549 cells with SIRT6 overexpression. (I) Representative western blot images and quantitative analysis of acetylated H3K9 levels in LPS-induced A549 cells with SIRT6 knockdown. (J) CHIP analysis was used to detect SIRT6 binding and H3K9 acetylation at the promoters of ERK1 and ERK2 in SIRT6-overexpression A549 cells. All experiments were conducted in triplicate. Data are shown as the mean ± SD. The horizontal lines above bars indicate comparisons between groups, ns: no significance, *p<0.05, **p<0.01.

The ERK1/2 pathway plays an important role in maintaining biological function 28. Earlier studies revealed ERK1/2 to be activated in ARDS 27. We assessed the expression of ERK1/2 and phosphorylated ERK1/2 (p-ERK1/2). Western blot analysis showed that LPS stimulation had no significant effect on the expression of total ERK1/2, but p-ERK1/2 levels increased after treatment. In addition, ERK1/2 and p-ERK1/2 levels were significantly lower in SIRT6-overexpressed A549 cells compared with the vector group, whereas A549 cells with SIRT6 knockdown showed a consistent increase in ERK1/2 and p-ERK1/2 levels (Figure 3A-D). qRT-PCR results also showed that overexpression of SIRT6 inhibited ERK1/2 mRNA levels (Figure 3E). To elucidate the mechanism of SIRT6 in regulating the ERK1/2 pathway, we performed Co-IP assays to clarify the interaction between SIRT6 and ERK1/2. The results showed that SIRT6 was unable to co-precipitate with ERK1/2 in SIRT6-overexpressed A549 cells when SIRT6 was pulled down (Figure 3F). Subsequently, we further evaluated the level of ERK1/2 acetylation in SIRT6-knockdown A549 cells when ERK1/2 was pulled down. Co-IP assays showed that the knockdown of SIRT6 increased ERK1/2 acetylation levels. In addition, ERK1/2 and SIRT6 failed to co-precipitate when ERK1/2 was pulled down (Figure 3G). Considering that SIRT6 is a nuclear histone deacetylase and directly regulates various gene expressions by deacetylating H3K9 in the chromatin, we detected the acetylated H3K9 (histone H3 lysine 9) level in A549 cells in which SIRT6 was overexpressed and knocked down. Western blot analysis showed that the overexpression of SIRT6 decreased acetylated H3K9 whereas the knockdown of SIRT6 increased it (Figures 3H and I). Previous research has demonstrated that SIRT6 interacts with the mitogen-activated protein kinase promoter 29. To explore a similar mechanism in A549 cells, we conducted a CHIP analysis to see whether SIRT6 regulates ERK1/2 signaling at the chromatin level. Increased SIRT6 binding at the promoter was related to lower H3K9 acetylation according to a CHIP assay (Figure 3J). These findings suggest that SIRT6 inhibits the expression of ERK1/2 signaling by deacetylating H3K9 on the promoters of ERK1/2 genes.

### SIRT6 alleviates inflammation, apoptosis, and TJ injury by inhibiting the ERK1/2 pathway and activating autophagy in LPS-induced A549 cells

**Figure 4:**
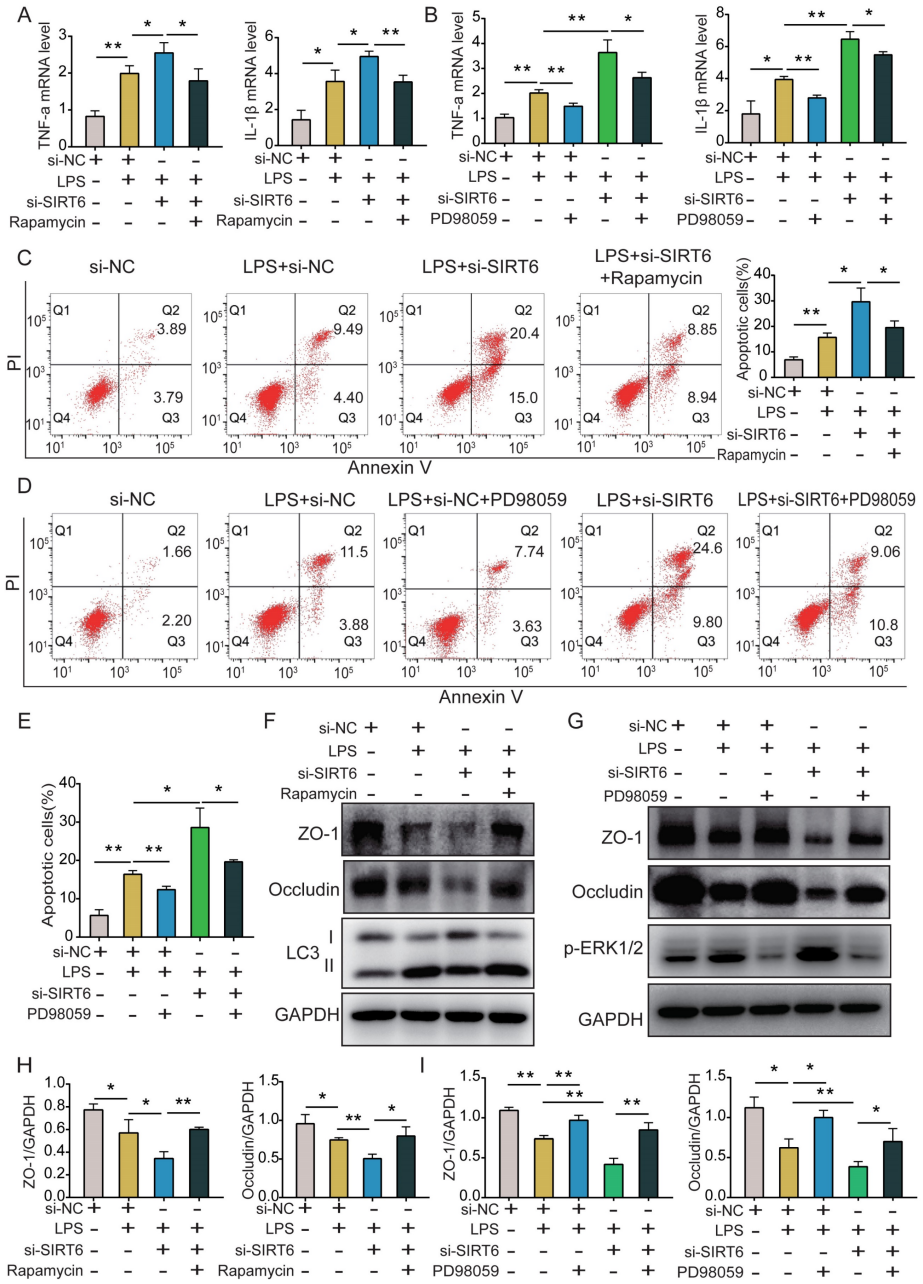
** SIRT6 alleviates inflammation, apoptosis, and TJ injury by inhibiting the ERK1/2 pathways and activating autophagy in LPS-induced A549 cells.** (A) The mRNA levels of TNF-a and IL-1β were tested by qRT-PCR in LPS-induced A549 cells after co-incubating with si-SIRT6 and rapamycin. (B) The mRNA levels of TNF-a and IL-1β were tested by qRT-PCR in LPS-induced A549 cells after co-incubating with si-SIRT6 and PD98059. (C) The proportion of apoptotic cells was detected by flow cytometry in LPS-induced A549 cells after co-incubating with si-SIRT6 and rapamycin. (D and E) The proportion of apoptotic cells was detected by flow cytometry in LPS-induced A549 cells after co-incubating with si-SIRT6 and PD98059. (F and H) Representative western blot images and quantitative analysis of TJ proteins (Zo-1, occludin) in LPS-induced A549 cells after co-incubating with si-SIRT6 and rapamycin. (G and I) Representative western blot images and quantitative analysis of TJ proteins (Zo-1, occludin) in LPS-induced A549 cells after co-incubating with si-SIRT6 and PD98059. All experiments were conducted in triplicate. Data are shown as the mean ± SD. The horizontal lines above bars indicate comparisons between groups, *p<0.05, **p<0.01.

To clarify the role of SIRT6-mediated autophagy and further explore whether SIRT6 protects against ARDS through ERK1/2 signaling, we used the ERK1/2 inhibitor PD98059, si-SIRT6, and the autophagy agonist rapamycin on A549 cells. qRT-PCR results showed that while si-SIRT6 significantly increased TNF-α and IL-1β levels in LPS-induced A549 cells, rapamycin and PD98059 reversed this effect (Figure 4A and B). Similarly, flow cytometry results showed that si-SIRT6 could significantly increase apoptosis after LPS treatment, and rapamycin and PD98059 reversed this effect (Figure 4C-E). Moreover, western blot results showed that si-SIRT6 significantly aggravated the LPS-induced decrease of TJ proteins, which was reversed after both rapamycin and PD98059 treatment (Figure 4F and G). Figure 4H and Figure 4I show the quantitative analysis of ZO-1 and occludin protein expression among the different groups.

### SIRT6 inhibitor OSS_128167 aggravates lung inflammation and suppresses lung autophagy *in vivo*

**Figure 5:**
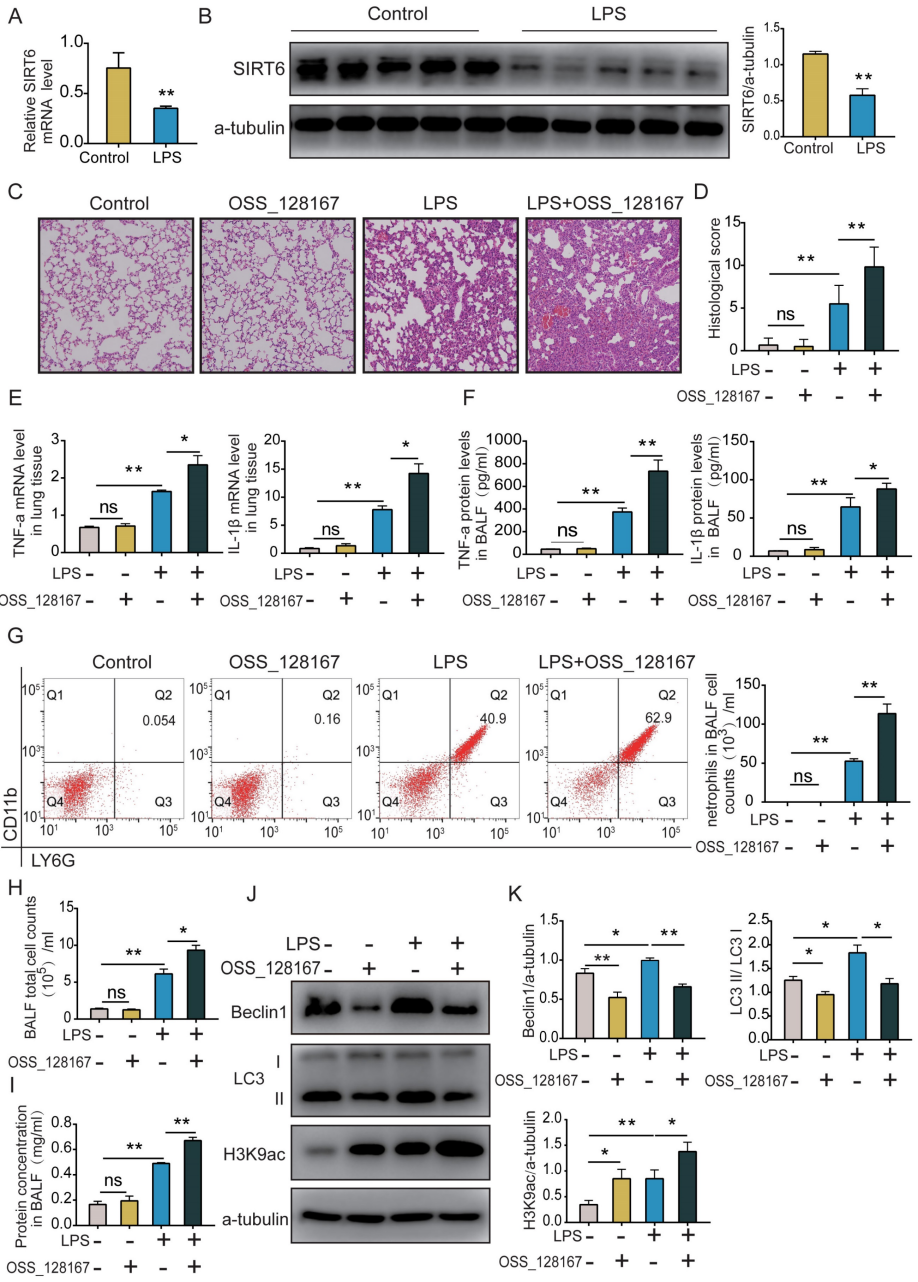
** SIRT6 inhibitor OSS_128167 aggravates lung inflammation and suppresses lung autophagy *in vivo*.** (A) mRNA levels of SIRT6 in lung tissue were tested by qRT-PCR. (B) Representative western blot images and quantitative analysis of SIRT6 protein in lung tissue. (C and D) The severity of mouse lung injuries was evaluated by HE staining and lung injury score. (E) mRNA levels of TNF-a and IL-1β in lung tissue were detected by qRT-PCR. (F) Protein levels of TNF-a and IL-1β in BALF were detected by ELISA. (G) Flow cytometric dot plots of neutrophils in the BALF, the total BALF cells were first gated on CD45 positive cells, and then gated on Ly6G and CD11b positive cells. (H) The count of total cells in BALF. (I) Protein concentrations in BALF were measured using BCA. (J and K) Representative Western blots images and statistical analysis of Beclin1, LC3, and H3K9ac proteins. Data are shown as the mean ± SD, n=6 for each group. The horizontal lines above bars indicate comparisons between groups, ns: no significance, *p<0.05, **p<0.01.

C57BL male mice were intratracheally instilled with LPS for 24 h. Lung tissues were collected, and the SIRT6 level was detected by qRT-PCR and western blot. These results showed that SIRT6 levels were significantly decreased in the LPS group compared with the control group (Figure 5A and B). Histological evaluation showed significant neutrophil infiltration, hyaline formation, septal thickening, and protein exudation after LPS administration. The SIRT6 inhibitor OSS_128167 significantly aggravated those injuries (Figure 5C and D). We measured the levels of inflammatory cytokines in lung tissue and BALF. qRT-PCR and ELISA results showed no differences between the OSS_128167 group and control group. However, in LPS-induced mice, OSS_128167 further increased TNF-α and IL-1β levels (Figure 5E and F). Neutrophils distinguished by flow cytometry (CD45**^+^**Ly6G**^+^**CD11b**^+^**) were the dominant inflammatory cells in the BALF of ARDS mice. OSS_128167 promoted LPS-induced neutrophil accumulation (Figure 5G). We found that the total number of BALF cells significantly increased in ARDS mice (Figure 5H). BCA results on the BALF showed that OSS_128167 significantly aggravated LPS-induced protein exudation (Figure 5I). Histones are substrates for SIRT6. The acetylation level of H3K9 was significantly increased in ARDS mice, and OSS_128167 further increased the acetylation level of H3K9. Autophagy in ARDS mice was increased, and OSS_128167 inhibited LPS-induced autophagy (Figure 5J and K). These results are consistent with *in vitro* findings, indicating that SIRT6 inhibition aggravates lung inflammation and inhibits lung autophagy.

### SIRT6 inhibitor OSS_128167 aggravates lung apoptosis and TJ injury *in vivo*

**Figure 6:**
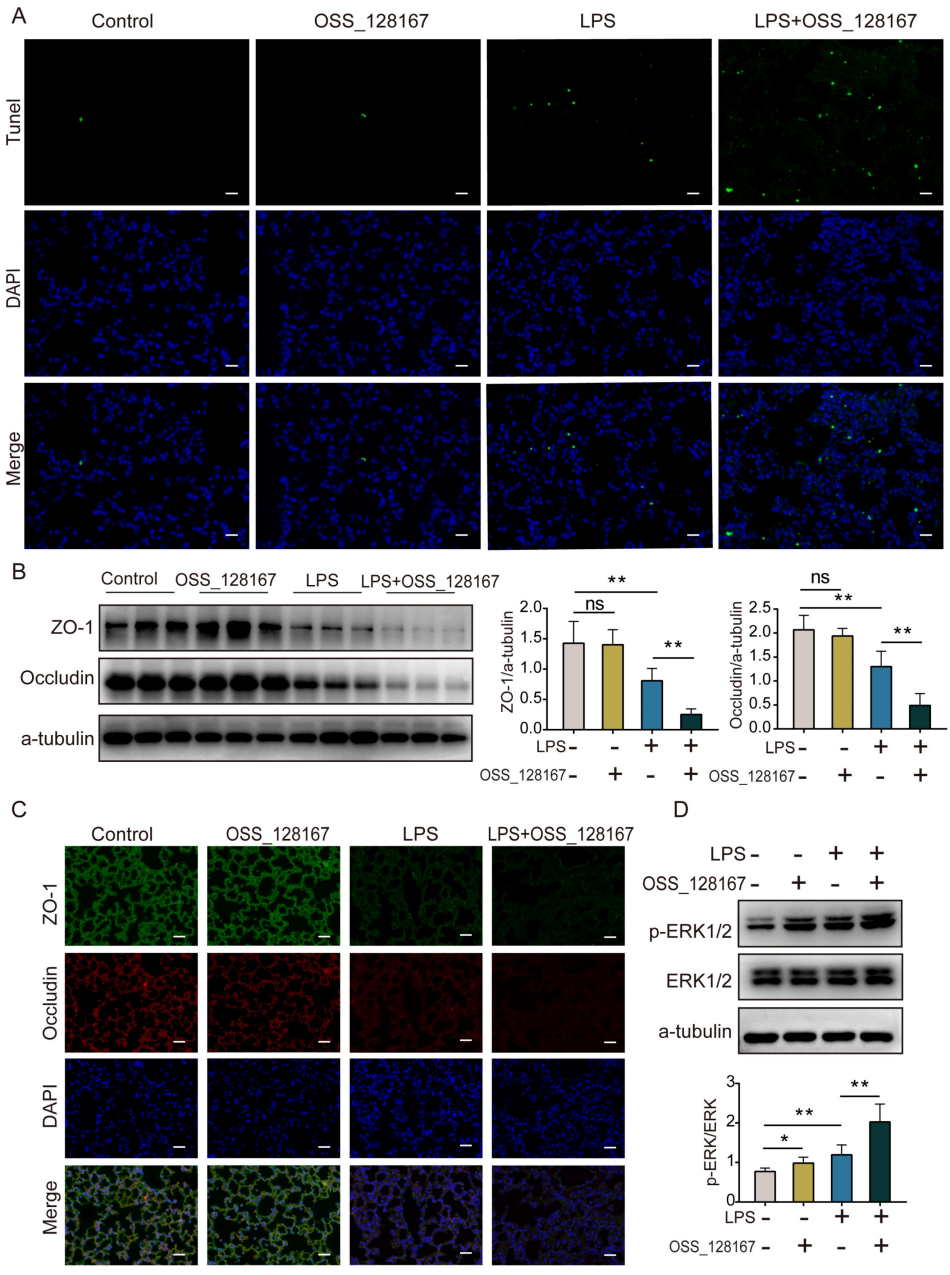
** SIRT6 inhibitor OSS_128167 aggravates lung apoptosis and TJ injury *in vivo*.** (A) Representative lung images of TUNEL assay (magnification, 400×), Scale bar=20μm. (B) Representative western blots images and statistical analysis of TJ proteins (ZO-1, occludin). (C) Representative immunofluorescence staining of TJ proteins (ZO-1, occludin) in lung tissue (magnification, 400×), Scale bar=20μm. (D) Representative western blot images and quantitative analysis of p-ERK1/2 protein. Data are shown as the mean ± SD, n=6 for each group. The horizontal lines above bars indicate comparisons between groups, ns: no significance, *p<0.05, **p<0.01.

The TUNEL assay showed that lung apoptosis increased in ARDS mice, and OSS_128167 further significantly increased LPS-induced apoptosis in mice (Figure 6A). Western blot and immunofluorescence showed that the expression of ZO-1 and occludin proteins was decreased in ARDS mice, and that the SIRT6 inhibitor OSS_128167 further aggravated the downregulation of ZO-1 and occludin proteins in LPS-induced ARDS mice (Figure 6B and C). Meanwhile, the ERK1/2 pathways were detected *in vivo*. Western blot results showed that p-ERK1/2 was increased in ARDS mice, and OSS_128167 further increased p-ERK1/2 levels in ARDS mice (Figure 6D). These results suggest that the SIRT6 inhibitor OSS_128167 further aggravates apoptosis and the loss of TJ proteins during ARDS.

## Discussion

SIRT6 is a histone deacetylase that regulates gene expression by deacetylating histones 30. It has shown protective effects in aging, cardiovascular diseases, nervous system diseases, cancer, and infectious diseases 17. Studies on SIRT6 in ARDS are few. Our research found that SIRT6 improved intercellular TJ protein damage, apoptosis, and inflammation in LPS-induced ARDS. Furthermore, in terms of molecular mechanisms, SIRT6 played a protective role by inhibiting ERK1/2 expression through the deacetylation of H3K9 and activating autophagy. These investigations uncovered new regulatory mechanisms of SIRT6 underlying LPS-induced ARDS, providing a theoretical basis for the development of targeted therapy for ARDS.

In ARDS, pulmonary edema is the main factor of poor prognosis of ARDS. The destruction of intercellular TJ proteins and apoptosis are the main causes of pulmonary edema 1. Maintaining the integrity of TJs and reducing apoptosis are both important for the prognosis of ARDS 31. Although SIRT6 has been shown to improve the expression of intercellular TJ proteins in hypertension and colitis 19, 32, its role in LPS-induced ARDS has not been elucidated. According to our work, the expression of SIRT6 was downregulated in LPS-induced ARDS, and we found that SIRT6 was critical for maintaining the integrity of the intercellular TJ proteins in LPS-induced ARDS. Western blot and immunofluorescence experiments demonstrated that the overexpression of SIRT6 significantly restored TJ protein expression and that knocking down SIRT6 and inhibiting SIRT6 further aggravated LPS-induced TJ protein loss. We demonstrated for the first time that SIRT6 plays a protective role in LPS-induced ARDS by improving intercellular TJs. In addition, our research also found that SIRT6 knockdown and a SIRT6-specific inhibitor increased lung inflammation and apoptosis, while the overexpression of SIRT6 attenuated LPS-induced inflammation and apoptosis. This is consistent with previous studies 19, 25 showing that SIRT6 protects against several illnesses by preventing inflammation and apoptosis. These results indicate that SIRT6 plays a protective role in the pathogenesis of ARDS.

Autophagy is the digestive system of cells and is considered one of the main pathways for the degradation and recycling of cellular components 33. It is unclear whether autophagy serves as a pro-survival or pro-death program, and the role of stress-mediated autophagy in ARDS is complicated 33, 34. However, a growing number of studies have indicated that autophagy activation may operate as a protective mechanism against LPS-induced ARDS. For example, in ARDS, RAB26 enhanced adherens junctional integrity in an autophagy-dependent manner 35. Exosomes from bone marrow mesenchymal stem cells reduced LPS-induced acute lung damage by regulating alveolar macrophage autophagy 36. Our study found that autophagy was activated in LPS-induced ARDS. We further found that the SIRT6 inhibitor OSS_128167 or the knockdown of SIRT6 suppressed autophagy, while the overexpression of SIRT6 increased autophagy. This is consistent with earlier research that found a connection between SIRT6 and autophagy 20, 37. The role of SIRT6-activated autophagy in LPS-induced lung injury is fascinating to us. Autophagy activation has been shown to improve TJ protein expression in Alzheimer's disease and peritoneal fibrosis 38, 39. Autophagy is also closely related to inflammation and apoptosis 11, 40. The role of SIRT6-associated autophagy in LPS-induced lung injury remains unclear. We co-incubated SIRT6 siRNA and rapamycin (a pharmacological agonist of autophagy) in LPS-induced A549 cells. The results demonstrated that rapamycin reversed the autophagy inhibition caused by SIRT6 knockdown in LPS-induced A549 cells, thereby attenuating the negative effects of SIRT6 knockdown on TJ proteins, apoptosis, and inflammation. Overall, our research found that SIRT6 could alleviate the above injury by enhancing autophagy. This also indicates that LPS-induced autophagy is a protective effect against external damage.

Meanwhile, we found that ERK1/2 is downstream of SIRT6. The ERK1/2 pathway has been demonstrated to be activated in ARDS and involved in regulating ARDS progression 41. We discovered that the overexpression of SIRT6 suppressed ERK1/2 and p-ERK1/2 levels, while SIRT6 knockdown in cells increased ERK1/2 and p-ERK1/2 levels. It has been previously reported that SIRT6 binds to ERK1/2 promoters and deacetylates histones in their promoter regions in mammals 29. To further clarify the regulatory mechanism of SIRT6 on ERK1/2 in ARDS, we show that SIRT6 knockdown increased acetylated H3K9 expression while SIRT6 overexpression had the reverse effect in A549 cells. Acetylated H3K9 expression was likewise increased by SIRT6 inhibitors in ARDS mice. Our result shows that SIRT6 bound to the promoter regions of ERK1/2, inhibiting ERK1/2 expression by deacetylating H3K9 at the promoter. Subsequently, we co-incubated SIRT6 siRNA and PD98059 (ERK1/2 inhibitor) in LPS-induced A549 cells. According to our findings, the ERK1/2 inhibitor PD98059 decreased the inflammatory cytokine levels in LPS-induced A549 cells with SIRT6 knockdown. ERK1/2 is a pro-apoptotic signaling molecule activated in response to DNA damage 42, 43. Our study shows that apoptosis exacerbated by SIRT6 knockdown in ARDS could be reversed by inhibiting the ERK1/2 pathway. Considering that the ERK1/2 pathway is involved in numerous cellular processes, it also plays an important role in maintaining the integrity of TJs. The ERK1/2 pathway is involved in TJ protein expression in kidney and colorectal cancer 44, 45. Our study shows that ERK1/2 inhibitors reversed TJ protein loss in LPS-induced A549 cells with SIRT6 knockdown. In mice, SIRT6 inhibitors also activated the ERK1/2 pathway, exacerbating LPS-induced TJ protein loss and inflammation. This is consistent with *in vitro* studies. This study demonstrates for the first time that SIRT6 improves intercellular TJs, inflammation, and apoptosis by inhibiting ERK1/2 pathways.

In conclusion, this study demonstrates that SIRT6 plays an important protective role in the regulation of inflammation, apoptosis, and TJ proteins in ARDS by inhibiting the ERK1/2 pathway and enhancing autophagy. The development of SIRT6 small molecule therapeutic agents may bring good news to the clinical treatment of ARDS. The limitations of this study are that the mechanism of SIRT6 in the regulation of autophagy and the correlation between SIRT6 and the prognosis of clinical patients need further study. We will continue to carry out relevant research in the future.

## Figures and Tables

**Figure 7:**
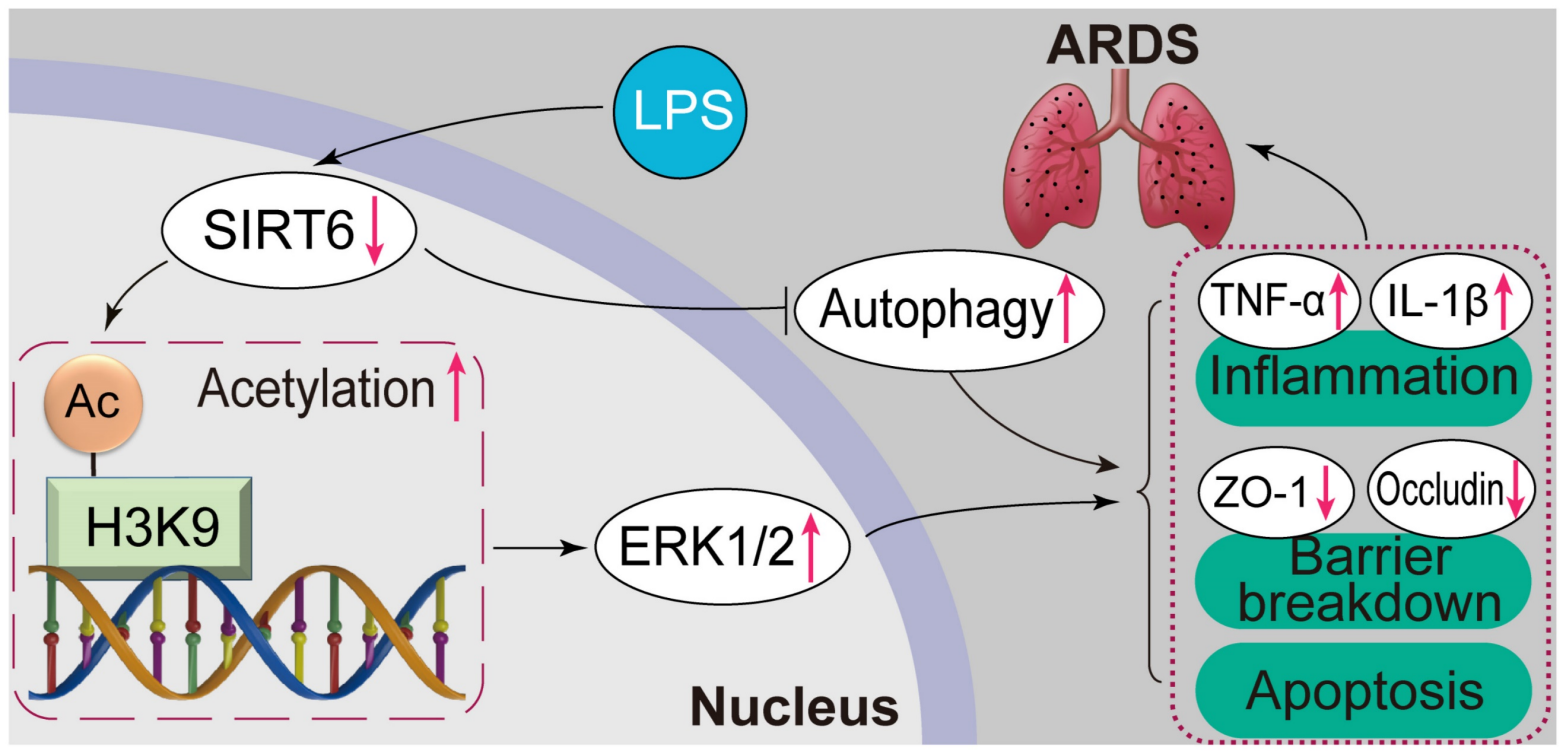
** Overview of the effects and mechanisms of SIRT6 in LPS-induced ARDS.** Under normal conditions, SIRT6 can activate autophagy and inhibit the expression of the ERK1/2 pathway. Under pathological conditions, the expression of SIRT6 was significantly down-regulated after LPS stimulation. On the one hand, SIRT6 reduction leads to increased H3K9 (histone H3 lysine 9) acetylation (H3K9ac) level in the ERK1/2 promoter, thereby promoting ERK1/2 gene transcription, and further exacerbating inflammation, apoptosis, and tight junction protein loss. On the other hand, SIRT6 reduction inhibits autophagy, which in turn exacerbates inflammation, apoptosis, and tight junction protein loss.

**Table 1:** Primers for qRT‐PCR

Genes	Species	Primer	Primer sequences
SIRT6	human	Forward Sequence	TGTGGAAGAATGTGCCAAGT
		Reverse Sequence	CTTAGCCACGGTGCAGAG
GAPDH	human	Forward Sequence	GGAGCGAGATCCCTCCAAAAT
		Reverse Sequence	GGCTGTTGTCATACTTCTCATGG
TNF-a	human	Forward Sequence	TCTTCTCATTCCTGCTTGTGG
		Reverse Sequence	GGTCTGGGCCATAGAACTGA
IL-1β	human	Forward Sequence	TTGACGGACCCCAAAAGAT
		Reverse Sequence	AGCTGGATGCTCTCATCAGG
ERK1	human	Forward Sequence	CTACACGCAGTTGCAGTACAT
		Reverse Sequence	CAGCAGGATCTGGATCTCCC
ERK2	human	Forward Sequence	TACACCAACCTCTCGTACATCG
		Reverse Sequence	CATGTCTGAAGCGCAGTAAGATT
SIRT6	mouse	Forward Sequence	CTCCAGCGTGGTTTTCCACA
		Reverse Sequence	GCCCATGCGTTCTAGCTGA
GAPDH	mouse	Forward Sequence	AGGTCGGTGTGAACGGATTTG
		Reverse Sequence	GGGGTCGTTGATGGCAACA
TNF-a	mouse	Forward Sequence	ATGTCTCAGCCTCTTCTCATTC
		Reverse Sequence	GCTTGTCACTCGAATTTTGAGA
IL-1β	mouse	Forward Sequence	GAAATGCCACCTTTTGACAGTG
		Reverse Sequence	TGGATGCTCTCATCAGGACAG
**Primer sequences for qRT-PCR analysis after CHIP analysis**			
ERK1	human	Forward Sequence	CTTTGTAAGGCCACAGCAGCAG
		Reverse Sequence	GCTGGCAGTAGGTCTGATGTTC
ERK2	human	Forward Sequence	TGCTGGTGAGGACTTGTGGA
		Reverse Sequence	TAGCACAGTACCTGCCACGA

## Abbreviations

ARDS: acute respiratory distress syndrome
LPS: lipopolysaccharide
SIRT6: sirtuin6
Tight junctions: TJs
FBS: fetal bovine serum
PBS: phosphate-buffered saline
DAPI: 4′,6-diamidino-2-phenylindole
PVDF: polyvinylidene difluoride
BALF: analysis of bronchoalveolar lavage fluid
DMEM: Dulbecco's modified Eagle's medium
ELISA: Enzyme-linked immunosorbent assay
TNF-α: tumor necrosis factor-alpha
Co-IP: co-immunoprecipitation
CHIP: chromatin immunoprecipitation
